# A Limited Opportunity: COVID-19 and Promotion of Advance Care Planning

**DOI:** 10.1089/pmr.2021.0013

**Published:** 2021-06-23

**Authors:** Kara B. Dassel, Gail L. Towsley, Rebecca L. Utz, Lee Ellington, Alexandra Terrill, Debra Scammon, Alycia A. Bristol, Amber Thompson, Melody Mickens

**Affiliations:** ^1^ College of Nursing, School of Medicine, University of Utah, Salt Lake City, Utah, USA.; ^2^ Department of Sociology, College of Social and Behavioral Sciences, School of Medicine, University of Utah, Salt Lake City, Utah, USA.; ^3^ Department of Occupational and Recreational Therapies, College of Health, School of Medicine, University of Utah, Salt Lake City, Utah, USA.; ^4^ Department of Marketing, David Eccles School of Business, School of Medicine, University of Utah, Salt Lake City, Utah, USA.; ^5^ Division of Physical Medicine and Rehabilitation, School of Medicine, University of Utah, Salt Lake City, Utah, USA.

**Keywords:** caregiving, end-of-life care, pandemic

## Abstract

***Background:*** Little is known about how COVID-19 has influenced the role of family caregivers in advance care planning (ACP).

***Objectives:*** To explore the experiences of family caregivers and ACP in the United States during the COVID-19 pandemic.

***Design:*** Exploratory sequential mixed-methods design of caregiver characteristics and pandemic response to ACP.

***Settings/Subjects:*** Family caregivers of care recipients with varied caregiving needs (dementia, mental illness, etc.).

***Measurements:*** Quantitative survey was done of fixed-choice questions of 82 caregivers. Semistructured qualitative telephone interviews were performed of a subsample of participants (*n* = 28).

***Results:*** Some (19%) of family caregivers revisited or updated advance directives of care recipients and/or had some type of contingency plan (33%) if they were to become ill. We identified three barriers caregivers faced during the pandemic that may have limited their engagement with ACP.

***Conclusions:*** Family caregivers need education regarding ACP and specific resources that can guide and support them through the process of ACP, for both themselves and care recipients.

Fifty-three million Americans provide care to family members, friends, and neighbors who are aging, chronically ill, and/or disabled.^1^ Caregivers provide medical, emotional, financial, and legal support.^2,3^ Their support occurs across all stages of illness, disability, or condition, but especially near end of life (EOL). High-quality EOL care emphasizes advance care planning (ACP), which is an ongoing process of communication and documentation (e.g., advance directive) that guides future medical care based on a shared understanding of a patient's goals, values, and preferences.^4^ The ACP process not only benefits patients, but also caregivers, by relieving caregiver burden (e.g., anxiety about future patient care needs, finances, and EOL decision making), improving subjective well-being, and decreasing anxiety.^5,6^

COVID-19 has amplified the need for patients and families to engage in ACP.^7,8^ In fact, some have suggested that the pandemic, which affects patients regardless of age, may be the impetus for families to engage in future health care planning, and will elevate the importance of ACP.^9^ Communication tools have been created to facilitate ACP.^10,11^ One portal reported a nearly fivefold increase in users and advance directive completion during the early months of the pandemic^12^; however, we know little about involving family caregivers in ACP conversations and documentation of care preferences during the COVID-19 pandemic. This brief report explores how caregivers engaged in ACP as they faced new demands associated with the COVID-19 pandemic.

## Methods

Using an exploratory sequential mixed-method design, we collected data with an electronically delivered survey^13^ that explored the experience of caregivers during the COVID-19 pandemic in terms of access to resources, respite, social isolation, stress and coping, and ACP. A subsample (*n* = 28) of the total sample of 82 participants (34% response rate) volunteered to be contacted to participate in semistructured telephone interviews. Surveys were collected from May through June 2020; telephone interviews were conducted with about one-third of the sample between July and September 2020. The institutional review board at the (BLINDED FOR REVIEW) approved study procedures.

Recruitment was done through social media and flyers were distributed by local/national organizations such as the Alzheimer's Association and Area Agencies on Aging. Eligibility criteria included 18 years or older and self-identifying as a caregiver (defined as an individual who provides care such as emotional support and/or assists with basic care tasks) to another person of any age with a mental and/or physical health condition or disability, without receiving pay for care.

Three fixed-choice items from the larger survey focused on ACP: (1) “Does the person you are caring for have an advance directive?” (definition of an advance directive was provided), (2) “Has it [advance directive] been updated since COVID-19?”, and (3) “Have you made a plan with the person you care for about what provisions for caregiving will be made should you become ill?” Descriptive statistics were used to analyze responses.

Two semistructured questions from the qualitative interview script focused on ACP: (1) “How has the spread of COVID-19 impacted discussions about EOL care with you and the person you provide care for?” and (2) “How comfortable or uncomfortable have you felt leading or participating in discussions about EOL care concerns with the person you care for and/or other family members?” Interviews were recorded and transcribed. Four members of the study team (BLINDED FOR REVIEW) engaged in initial coding, subsequently reviewed coding, and added and revised codes to identify overarching domains. Quantitative and qualitative data were analyzed separately and integrated using a contiguous approach.^14^

## Results

Caregivers (*N* = 82) were predominantly female (85%), Caucasian (94.1%), non-Hispanic (66.1%), had at least a college education (74.0%), married (78%), and older (64% over the age of 50 years). Participants primarily provided >20 hours of care a week (53%) for a spouse/partner (27%) or parent/parent in-law (15%) and coresided with them (73%). Sixty-three percent said that their care recipient had an advance directive; 19% had updated it since the start of the pandemic. One-third (33%) had made a contingency plan, should they become sick from COVID-19 and be unable to provide care. Contingency plans consisted of enlisting additional family help, sending children to another household, frequent telephone check-ins, use of in-home paid services, and temporary or permanent placement in long-term care.

Table 1 illustrates three domains related to ACP and COVID-19 based on responses from a subsample of participants who completed interviews (*n* = 28): (1) *Interruption of Care Plans* refers to caregivers experiencing the sudden halt of planned care transitions and loss of needed supports, services, and resources; (2) *Focused on the Present* refers to how caregivers focused on the immediate needs of the care recipient rather than engaging in future planning; and (3) *Navigating without a Compass* refers to the lack of education about ACP resources, supports, or guidance for caregivers.

**Table 1.** Barriers to Advance Care Planning During the COVID-19 Pandemic

Domain	Context	Exemplar quotes
*Interruption of Care Plans*	Before the pandemic a family moved across the country to support the VA rehabilitation services for their 38-year-old son who had a sudden stroke. They struggled as the active services ceased. A woman who is the primary caregiver for her husband with dementia, needed to have hip surgery.	*He was doing a lot of therapy. And that all stopped, except for the type that comes in over the phone, and over the computer. I think the hardest thing, really, is finding interesting things to do in the apartment, that [he] can do and enjoy. That—I suspect that's the—that's probably the hardest thing.* ….well, it was probably about two months into the pandemic I had to have a hip surgery… in addition to trying to take care of my husband, who has Alzheimer's…and so for the last month we've been trying to get him placed into a home, and it's just been hard because I've had to rely on a lot of other people, all my kids and my neighbors…And the thing that made it even additionally, yeah, hard was the fact that we had him an adult daycare to start going to a couple times a week, and he went once, and then the pandemic shut everything down, so then there was nothing for me. There was no place that I could turn to that I could have him go to…
*Focused on the Present*	A woman caring for her husband with dementia is focused on her husband's day-to-day needs. Participant and her husband moved to be 10 minutes away from where their son who has autism was just starting university. He was living on campus and doing well until he had to move in with them due to COVID-19.	*Well, I guess I don't really look too far in the future. At some point, my husband's probably going to need home health care, or hospice care…Focus on the day to day: I guess, with COVID, I have to keep a little bit of a closer watch on him. If the doorbell rings, he'll go to the doorbell and forget that he shouldn't answer the door or let people in, so a greater watch with it. I monitor his health really closely. I nudge him a lot more about using the walker.* We have no backup care. I mean, we don't even have care that like if—like we were talking about possibly going away ourselves, just for like two days, just because it's been so much, and I've got this vacation time. And we couldn't even get away for a weekend, because I realized, I was like, I don't feel comfortable like leaving [son] I don't even think I'd feel comfortable, honestly, leaving him for overnight, because I don't trust that he can make good decisions, whether he'd have people over, or he would go out and do something that we wouldn't necessarily think was safe social distancing behaviors.
*Navigating without a Compass*	A woman caring for her husband discussed how COVID-19 has not urged them to complete an advance directive. A daughter who is the primary caregiver for her mom discusses how they completed ACP years before COVID-19 and did not feel any need to revisit them due to the pandemic.	*No. I don't think it's occurred to us, to [complete an advance directive]. I think we're just hoping for the best possible outcome. And, you know, compared to where we were in February and the first part of March, where everybody was just stopped, terrified, because there weren't any treatments, I think we've all seen so many people recover, and we've seen so many variations of what the illness is for so many people, that we're just hoping that we would get to be in the we've got good genes category, and it's going to go okay. What else are you going to do? I mean, what are you going to do? Hope for the best.* Yeah. Yeah. My mom and I actually, you know, got her, you know, put all the finances in order before COVID. Trust, living will, medical directives, signed, sealed, delivered all of that before COVID. So, from like a logistical perspective, like, I feel like I know what to do. I don't feel like we're close to that but I don't—that hasn't spurred conversation because from my vantage point, like, it's pretty buttoned up and that's been something that we prioritized before COVID.

Participant Interview Responses.

ACP, advance care planning.

## Discussion

Caregiver participants were more likely to report that their care recipient had an advance directive (63%), compared with national estimates, where only about one-third of adults have an advance directive.^4^ Not only did our sample have a high completion rate of advance directives, about a fifth of participants' reported that they revisited the documents during the COVID-19 pandemic. Furthermore, a third of caregivers reported that they had developed a contingency plan if they were to become ill or needed to quarantine. These results suggest that the pandemic spurred incremental change in some families in terms of completing or revising ACP in terms of advance directives and/or contingency planning. Our qualitative findings explain three potential barriers that caregivers faced during the COVID-19 pandemic that may have limited their engagement with ACP.

First, we refer to pauses, reversal, or (dis)continuation of services as “Interruption of Care Plans,” and identify as a barrier impeding caregivers' ability to carry out earlier-established care plans, and limiting options for any future care planning. COVID-19 resulted in the closing or modification of services of child or adult day centers, meals, transportation, etc. In addition, some caregivers who were in the process of transitioning a care recipient to a different residential option (e.g., group home, long-term care) were faced with the decision of whether to continue with the transition, whereas others had to consider removing a care recipient from residential environments due to COVID-19 risks. Although this barrier is likely unique to COVID-19, it demonstrates the importance of having or making contingency plans for caregiving.

Second, COVID-19 forced caregivers to *Focus on the Present*, which likely is a barrier to ACP in general, however, COVID-19 amplified this dynamic and provides a potential explanation for why only a small proportion revisited ACP or discussed possible contingency plans should a caregiver become ill. Caregivers provide ∼20 hours of care per week and often juggle competing demands leaving little time or opportunity to focus on matters beyond those required for basic day-to-day functioning, including their own health, which places them at risk to be unable to continue caregiving duties.^15^ With the closing of essential services, physical distancing, and quarantining, caregivers were isolated from their support networks and often had to address all of the patient's physical and emotional needs, while managing additional infection control precautions. To overcome focusing on the present, caregivers may benefit from having access to quick and simple tools that guide them through the most essential points for ACP conversations. Such tools, especially if they are user friendly and accessible through self-administered workbooks or apps, may help demystify the ACP process and make it more convenient and accessible.

Third, the domain of *Navigating Without a Compass* identified lack of knowledge as another barrier to ACP. Most had completed important preliminary forms of ACP (e.g., advance directive, establishing funeral plans), but were less likely to have ongoing conversations to explore personal values, life goals, and preferences regarding future medical care. ACP should include specific discussions about long-term care, goals of care, and EOL management.^16,17^ To achieve the full benefit of ACP, more education is needed, where caregivers are informed about the importance of engaging in comprehensive ongoing conversations about future care preferences and plans, and empowered to be part of the decision-making process with and sometimes on behalf of the care recipient. Although caregivers in this study did not specifically speak of how COVID-19 hospitalization options (e.g., use of a ventilator) may challenge previously rejected care choices, this should be explored in future studies.

This study has both strengths and limitations. Strengths include the timeliness of capturing caregiver experiences during the COVID-19 pandemic, including caregivers of care recipients with varied medical conditions and of various ages, and our mixed methods approach. Limitations include the homogeneous nature of our larger sample (*N* = 82) of primarily White, well educated, female caregivers, and our inability to link participants' qualitative and quantitative data due to the anonymity of the survey questions. Our study survey was electronic and, therefore, may have limited participation from caregivers of lower socioeconomic status who lack access to technology. Our study also collected data during the summer of 2020 when there was a wane in the COVID-19 pandemic positive cases and participants may have been feeling more optimistic about the eradication of COVID-19 and the return to “normalcy.”

## Implications

Existing resources describe the importance of ACP for general populations,^18–20^ for disease-specific contexts,^16,21^ and for specific environments (e.g., nursing homes).^22^ Health care providers, community-based aging agencies, and long-term care centers should be aware of these resources, and help disseminate them to the general population. Existing campaigns, such as Dementia Friendly America,^23^ Age-Friendly Health Care,^24,25^ and President Joe Biden's priority to create a caregiving workforce,^26^ are examples of platforms that may be used to distribute ACP education and outreach. In addition, public health agencies such as the Centers for Disease Control have a rare opportunity to further promote (beyond some individuals' natural reaction to revisit plans as in response to a disaster) ACP, rather than just focusing on precautions, as part of the COVID-19 safety protocol measures.

In conclusion, although the pandemic has been touted as an opportunity to elevate the importance of ACP,^10,11^ among family caregivers, we observed only a moderate increase in the level of ACP engagement. Our results identified three barriers that may impede caregiver engagement in ACP. Although one of these barriers may be unique to COVID-19, it points up the value of contingency planning. All three barriers can be addressed by providing specific education and tools to guide caregivers through the process of ACP, in a way that emphasizes the need for ongoing conversations, and help achieve EOL experiences that match one's values, preferences, and goals for care.

## Abbreviations Used

ACP: advance care planning
EOL: end of life
